# In-Hospital Diagnosis of Tricuspid Papillary Muscle Rupture in an Asymptomatic Patient after Blunt Chest Trauma

**DOI:** 10.1155/2019/1890640

**Published:** 2019-05-09

**Authors:** C. S. Nabzdyk, M. B. Tabrizi

**Affiliations:** ^1^Department of Anesthesia, Critical Care and Pain Medicine, Massachusetts General Hospital, Harvard Medical School, Boston, MA, USA; ^2^Department of Surgery, Massachusetts General Hospital, Harvard Medical School, Boston, MA, USA

## Abstract

Tricuspid papillary muscle rupture after blunt chest trauma is an infrequent injury that often remains undiagnosed until patients become symptomatic months to years after the trauma occurred. It is imperative to diagnose patients early with this condition in order to optimize chances of successful recovery and avoidance of sequelae of long-term tricuspid regurgitation such as atrial fibrillation and right heart failure. Here we describe a case of a 58-year-old man involved in a motocross accident suffering amongst other injuries extensive bilateral rib fractures, hemopneumothoraces, and asymptomatic anterior tricuspid papillary muscle rupture. In addition, a review of the literature and an approach for the workup of trauma patients at risk for blunt cardiac injury are provided.

## 1. Case Description

Our patient is a 58-year-old male with past medical history of hypertension and distant right femur fracture who was involved in a motocross accident suffering left- (1st-12th) and right-sided (7th, 9th, and 12th) rib fractures with bilateral hemopneumothoraces requiring bilateral chest tube placements. The patient also suffered a right intertrochanteric and a peri-implant femur fracture as well as a nondisplaced ulnar styloid process fracture. The patient was transferred from an outside hospital for further care. Upon arrival the patient was remarkably asymptomatic, in normal sinus rhythm, hemodynamically and respiratory stable, maintaining an O_2_ saturation between 95% and 100% with minimal supplemental O_2_ via nasal cannula. The patient denied significant chest pain or shortness of breath despite his significant injury burden. ECG findings showed some ST wave abnormalities suggestive of early repolarization. An initial troponin level of 0.15 normalized within 24h of admission. Given the patient's injury pattern and troponin leak in the absence of known coronary artery disease (CAD), congestive heart failure (CHF) pulmonary embolism (PE), or shock, a formal TTE was obtained. TTE revealed severe tricuspid regurgitation secondary to flail anterior tricuspid valve leaflet with preserved right ventricular geometry and systolic function. The cardiac surgery team recommended outpatient follow-up for elective repair in two months with repeat TTE.

The femur fracture was repaired on hospital day two under general anesthesia and the patient recovered well. The chest tubes could be sequentially removed without recurrence of pneumothoraces over the course of the admission. On hospital day three the patient experienced an episode of atrial fibrillation with rapid ventricular response that responded well to a single 5 mg intravenous bolus of metoprolol. After a few hours the patient converted back to normal sinus rhythm, in which he remained until the day of discharge on hospital day seven. The patient continued to deny any palpitations, shortness of breath, or radiating chest pain. By the time of discharge, the patient was able to ambulate, and his pain was well controlled with oral analgesics. Unfortunately, by one year after hospital discharge, the patient had not followed up with regard to his newly diagnosed tricuspid regurgitation.

## 2. Discussion

Tricuspid papillary muscle rupture is an infrequent complication from blunt chest trauma including falls, car and riding accidents [1–10]. Pedestrians struck by vehicles appear to sustain the most severe injuries when compared to other trauma mechanisms [11].

Clinical presentation, the combination of severity of associated injuries, ECG findings, and troponin levels should guide triage and subsequent workup of cardiac contusion including utilization of echocardiography [12–14]. In a study of 94 trauma patients the use of troponin levels alone did not appear to be an improved method to diagnosis of blunt cardiac injury in hemodynamically stable patients due to low sensitivity as well as low predictive values [15]. Also there was no association of myocardial contusion cell injury and late patient outcome when troponin levels and other conventional markers were considered [15]. Of note, none of the trauma patients without cardiac injury had elevated levels of troponin T [15]. A subsequent study including 115 trauma patients concluded that the combination of ECG and troponin I reliably identified the presence or absence of significant blunt cardiac trauma [16]. Accordingly, the investigators recommended close monitoring for at least 24 hours for patients with an abnormal ECG and elevated troponin I. In contrast, patients with a normal admission ECG and troponin I could be safely discharged in the absence of other injuries [16]. In this dataset, the combination of abnormal ECG and elevated troponin I yielded 100% sensitivity, 88% specificity, and 62% positive and 100% negative predictive value [16]. A larger follow-up study with 333 trauma patients largely confirmed the findings of the previous study [16]. In it the combination of abnormal ECG and elevated troponin I yielded 100% sensitivity, 71% specificity, and 34% positive and 100% negative predictive value for the detection of clinically significant blunt cardiac injury [16]. This study also identified three risk factors: Injury Severity Score (ISS) > 15, skeletal trauma, spinal, pelvic, major extremity fracture, and history of cardiac disease [16].

A separate study involving 187 trauma patients found a correlation between troponin I levels and risk of arrhythmias and acute reduction in left ventricular ejection fraction (LVEF) [17].

The Eastern Association for Surgery of Trauma guideline update from 2012 summarized the following: the combination of normal ECG and troponin I rules out blunt cardiac injury; normal ECG and abnormal troponin I warrant admission to a monitored setting; and persistent new arrhythmias and hypotension warrant echocardiographic evaluation [18]. When compared directly, transthoracic echocardiography (TTE) provided a lower diagnostic yield in severe blunt chest injury than transesophageal echocardiography (TEE) [19–22]. Some authors proposed cardiac troponin level measurements as a screening test prior to echocardiographic workup and data suggests that severity of cardiac arrhythmias correlates with cardiac troponin levels [17, 23]. Moreover, even in the absence of clinical evidence of cardiac involvement, troponin leaks may be associated with worse outcomes in patients with blunt chest trauma [13]. Despite these insights, it is believed that cardiac contusions and associated structural cardiac injuries remain underdiagnosed [24]. In fact, in several reported cases, symptom onset has been subtle over a period of months to years [3, 6, 9]. A recent case series reported a median delay of almost eight years between date of trauma and surgical repair of tricuspid papillary muscle rupture [1]. At the time of surgery, all patients suffered from severe tricuspid valve regurgitation. In comparison, a previous case series reported a median delay of 17 years from trauma to surgery [7]. While patients in both series recovered well from surgery, authors recommend early surgical repair in order to preserve right ventricular function, to allow for tricuspid repair rather than replacement, and possibly to maintain sinus rhythm in more patients [1, 7].

Acute, severe shock as a result of tricuspid papillary rupture after blunt chest trauma appears to be less common, contributing to the delayed diagnosis of tricuspid papillary injury in the context of blunt chest trauma. Clinical acuity seems to correlate with the extent of papillary injury. One case reported a need for emergency surgery in a young male patient involved in a car accident with rupture of all three papillary muscles of the tricuspid valve [25]. Another instance was reported in a young female after a riding accident with complete rupture of the posterior papillary muscle [8].

Given the usually insidious presentation of acute tricuspid papillary muscle rupture, we recommend a heightened suspicion, even in asymptomatic patients with high force blunt chest trauma, and initiation of echocardiographic evaluation. While TEE has been shown to be superior to TTE in diagnosing cardiac contusion-associated injuries in older studies, more recent studies are missing to account for improved imaging qualities that modern TTE devices may offer [19, 22]. Echocardiographic workup may be particularly indicated, if new ECG findings are present and cardiac troponin levels are elevated, especially in the absence of CAD, PE, or CHF, as it was the case in our patient.

This case illustrated that structural cardiac injury can be present even in the absences of major ECG, troponin I, or hemodynamic abnormalities and that echocardiographic evaluation is reasonable, if clinical suspicion is high.
